# Radio frequency identification technology reduce intravenous thrombolysis time in acute ischemic stroke

**DOI:** 10.1371/journal.pone.0288207

**Published:** 2023-07-19

**Authors:** Yixiong Zhang, Yingxia Jing, Yimin Zhu, Tao Jiang, Xianyi Tang, Weichen Yi

**Affiliations:** 1 The First People’s Hospital of Huaihua, Huaihua City, China; 2 Poisoning Research Laboratory, Institute of Emergency Medicine, Hunan Provincial People’s Hospital, The First Affiliate Hospital of Hunan Normal University, Changsha City, China; Foshan Sanshui District People’s Hospital, CHINA

## Abstract

**Purpose:**

To inspect whether time management with radio frequency identification technology (RFID) reduces symptom onset-to-intravenous thrombolysis time (OTT) in acute ischemic stroke (AIS).

**Methods:**

In the retrospective study, patients with AIS, transferred by Emergency Medical Services (EMS) to Hunan Provincial People’s Hospital between September 2019 to June 2022, divided into three groups, as traditional group, in-hospital RFID group and whole process RFID group. Baseline characteristics and time metrics were compared.

**Results:**

After the whole emergency process applied with RFID time management, Door to intravenous thrombolysis time (DNT) was reduced from 125.00±43.16 min to 32.59±25.45 min (F = 121.857, p<0.001), and OTT was reduced from 235.53±57.27 min to 144.31±47.96 min (*F* = 10.377, p<0.001).

**Conclusions:**

Time management with RFID is effective in reducing OTT in AIS patients with thrombolysis treatment.

## 1. Introduction

The incidence rate of ischemic stroke in China is 17.7%, twice the international rate [1]. Time is an important factor in the emergency care of patients with Acute Ischemic Stroke (AIS) [2]. Every one-minute delay in the patient’s AIS emergency care procedure can reduce the patient’s brain cells by 1.9 million [3]. It is recommended that intravenous thrombolytic therapy should be administered within 4.5 hours after the onset of AIS [4]. The first 60 minutes from symptom onset to intravenous thrombolysis (OTT) is the golden window in which to obtain medical treatment [4]. A multidisciplinary collaboration and continuous process optimization was proved to shorten intravenous thrombolysis time [5]. However, it depend on a precise time management, which could identify delays in workflow of AIS care, leading to better multidisciplinary and continuous optimization. The Hunan Provincial People’s Hospital has 2,000 beds and an annual inpatient population of 20,000. To provide AIS patients with intravenous thrombolysis treatment (IVT) in the shortest time possible after symptom onset, the Hospital established a stroke center in March 2019 based on the Guidelines for Early Management of Patient With AIS [6]. In September 2020, the stroke center introduced RFID technology to automatically record time points in the emergency care process of AIS patients, which provided an empirical basis for identifying causes in treatment delay for continuous quality assessment and improvement. In November 2021, the Hospital extended the RFID time management system to include the Emergency Medical Services (EMS) ambulance phase and record time points of both the pre-hospital and in-hospital phases of the emergency care procedure. The current study compares OTT before and after the introduction of the RFID technique, thereby exploring the effect of the RFID system on AIS emergency time management and patient hospital outcomes.

## 2. Methods

### 2.1 RFID installation and time points acquisition

**Fig 1:**
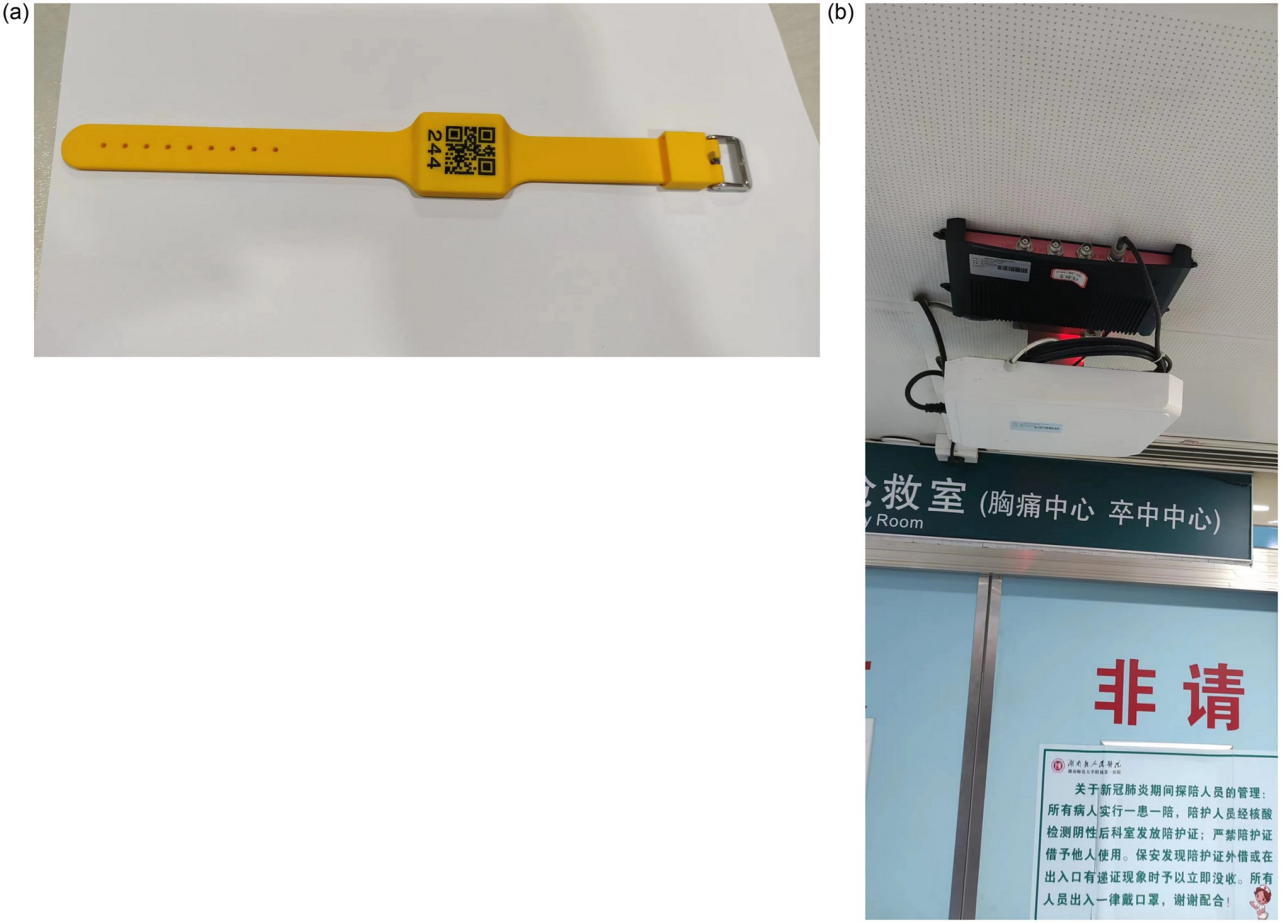
RFID radio frequency identification device including a, wristband; b, reader.

RFID is a radio frequency identification technology comprising mainly of two parts: a tag (a wristband) and a reader. The tag stores information, and the reader identifies the tag through radio frequency signals. The reader reads and decodes the information stored on the tag and sends it to a central information system for data processing. The RFID wristband is used as the tag (Fig 1a) stored for use at the triage desk. The reader (Fig 1b) is installed at various key nodes in the emergency care workflow as the AIS patient passes through the emergency care process such as the entrance of the emergency room, the computed tomography (CT) room, the interventional surgery room, the intensive care unit (ICU), etc. As the patient passes through each entrance, the reader automatically identifies the tag and records the patient’s time of arrival. Subsequently, the RFID technique was extended to include the pre-hospital phase by installing a RFID reader at the entrance of the ambulance’s patient compartment.

The emergency care procedure before and after the introduction of RFID is outlined below:

#### Traditional time management without RFID (September 2019 to August 2020)

(1)Patient calls for help. The EMS paramedic records the symptom onset time manually; the time of call is recorded automatically by the EMS telecommunications system. (2) Ambulance arrives on scene and the patient is transferred to the ambulance. The EMS paramedic records the ambulance’s arrival time manually. (3) Ambulance arrives at the hospital. Patient enters the emergency department for early diagnosis and treatment. Electrocardiogram examination is performed, and blood sample collected. The time is recorded by the triage nurse manually. (4) Patient enters the CT room. Time is recorded automatically as the technician logs on the Picture Archiving and Communication System (PACS) to initiate the CT examination. (5) Stroke physician makes decision on thrombolysis after the CT scan. Time is recorded automatically when medical staff logs the record in the electric medical record system.

#### In-hospital RFID time management (September 2020 to October 2021)

(1)Patient calls for help. The EMS paramedic records the symptom onset time manually. The time of calling for EMS is recorded automatically by the EMS telecommunications system. (2) Ambulance arrives on scene. Patient is transferred to the ambulance. Time of the ambulance’s arrival is recorded by the EMC paramedic manually. (3) Ambulance arrives at the hospital. Electrocardiogram examination is performed, and blood sample collected. The attending medical staff applies a RFID wristband on the patient. Patient is transferred to the emergency room for early diagnosis and treatment. Patient’s time of arrival is recorded automatically by the RFID reader at the entrance of the emergency room. (4) Patient enters the CT room. Time is recorded automatically by the RFID reader at the entrance of the CT room. (5) Stroke physician makes decision on thrombolysis after the CT scan. Time is recorded automatically when the medical staff logs the record in the electric medical record system.

#### The whole process RFID time management (November 2021 to June 2022)

(1)Patient calls for help. EMS paramedic records the symptom onset time manually. Time of calling for EMS is recorded automatically by the EMS telecommunications system. (2) Ambulance arrives on scene. EMC paramedic applies a RFID wristband on the patient. Time is recorded automatically by the RFID reader at the entrance of the patient’s compartment of the ambulance when patient is transferred into the ambulance. (3) Ambulance arrives at the hospital. Electrocardiogram examination is performed, and blood sample collected. Time is recorded automatically by the RFID reader at the entrance of emergency room when the patient enters the emergency room. (4)Patient enters the CT room. Time is recorded automatically by the RFID reader at the entrance of the CT room when patient pass through the entrance of the CT room. (5)Stroke physician makes decision on thrombolysis after the CT scan. Time is recorded automatically when the medical staff registers the record in the electric medical record system.

### 2.2 Patients selection

The data for the study came from all AIS cases from the Hunan Provincial People’s Hospital from September 2019 to June 2022 based on the following inclusion and exclusion criteria:

The inclusion criteria are:

(1)All patients conform to the diagnostic criteria according to the AHA/ASA guidelines [6] as follows: (a) acute onset; (b)with symptoms or signs of neurological deficits; (c)confirmation of AIS on brain CT/MRI; (d) exclude cerebral hemorrhage and non-vascular causes.
(2)All patients had potential indication of intravenous thrombolysis.
(3)Time of symptom onset to arrival is less than 4.5 hours.
(4)All patients arrived at the hospital by EMS.

The exclusion criteria are:

(1) Patient is under 18 years of age.
(2) Contraindications to intravenous thrombolysis, or thrombolysis received after 6 hours from symptom onset.
(3) In-hospital stroke.
(4) Stroke after resuscitation.
(5) Patient has received IVT outside the hospital.
(6) Patient’s head imaging was performed outside the hospital.
(7) Patient’s medical information is significantly incomplete.

The eligible cases were divided into three groups according to the stage in which RFID is implemented in the patient’s emergency care process: the traditional group before the implementation of the RFID technology (i.e., cases from September 2019 to August 2020), the in-hospital RFID group where the RFID technology is implemented at the in-hospital phase (i.e., cases from September 2020 to October 2021), and the whole process group when the RFID technology is extended to include the pre-hospital phase (i.e., cases from November 2021 to June 2022).

### 2.3 Data collection

The baseline data collected include the patient’s demographic characteristics, medical history, method of admission, clinical characteristics, treatments, and hospital patient outcomes as in-hospital mortality (Patients with deteriorating conditions who voluntarily abandoned treatment were treated as in-hospital death), intracerebral hemorrhage, hospital stays, NIHSS scores at discharge, mRS of discharge. In addition, the time metrics of the emergency care process were collected, including the onset to call time (OCT), call to ambulance on scene time (CAT), Ambulance on scene-to Door time (ADT), door to CT room (DCT), CT to IVT (CTT), symptom onset to door time (ODT), door to needle for thrombolysis time (DNT), OTT, etc. Data were obtained from the hospital’s electronic medical record system. Pre-hospital data were obtained from the EMS registry. Trained hospital personnel collected the data based on previously designed data collection forms.

### 2.4 Statistical analyses

Normally distributed measures are expressed as mean ± standard deviation, and non-normally distributed measures are presented as median and interquartiles. Categorical data are expressed as frequency or percentages. Differences between groups of normally distributed quantitative data were compared using the one-way analysis of variance (ANOVA) test. Differences among groups of non-normally distributed data or qualitative data were compared using the Mann-Whitney test. Differences among groups of categorical data were examined using the chi-square test. The statistical procedures were performed in the SPSS 22.0 statistical software, and p<0.05 was considered statistically significant.

### 2.5 Ethical considerations

The study was reviewed by the Ethics Committee of the Hunan Normal University. Informed consent was not obtained because the study is a retrospective analysis and all personally identifiable information was concealed in the statistical analytical process.

## 3. Results

### 3.1 Patients’ baseline information

From September 2019 through June 2022, a total of 338 cases consistent with an AIS diagnosis were transferred to the Hospital among which 5 cases were in-hospital stroke, 87 cases had symptom onset to IVT time greater than 4.5 hour, 8 cases were wake up stroke and 35 cases had significantly incomplete medical records. Subsequently, a total of 203 cases were enrolled in the study, including 134 males and 69 females, with an average age of 64.55±11.40. The grouping of enrolled cases and baseline information are shown in Table 1. There were no statistical differences in the demographic information among the three groups, except for the higher NIHSS score of the whole process RFID group than the other two groups (Table 1).

**Table 1:** Comparison of demographic characteristics and clinical characteristics among the three groups.

Demographic characteristics	Traditional Group (N = 30)	In-hospital RFID Group (N = 144)	Whole process RFID Group (N = 29)	*F/z/χ* ^*2*^	P
Gender, male,yes,n(%)	21(70%)	91(63.2%)	22(75.9%)	1.976	0.372
Age (years)*	67.13±9.16	64.53±11.29	61.97±13.61	1.525	0.220
Smoke, yes,n(%)	16(53.3%)	84(58.3%)	16(55.2%)	0.307	0.858
Hypertension, yes,n(%)	21(70%)	100(69.4%)	19(65.5%)	0.192	0.909
Coronary heart disease, yes,n(%)	17(56.7%)	87(60.4%)	16(55.2%)	0.362	0.834
Diabetes mellitus, yes,n(%)	15(50%)	82(56.9%)	13(44.8%)	1.676	0.433
Atrial fibrillation, yes,n(%)	9(30%)	55(38.2%)	6(20.7%)	3.587	0.166
Cerebrovascular disease, yes,n(%)	3(10%)	12(8.3%)	2(6.9%)	0.186	0.911
Clinical characteristics					
Systolic pressure (mmHg)*	145.03±22.88	147.58±22.62	154.83±28.39	1.469	0.233
Diastolic pressure (mmHg)*	81.20±13.07	86.06±14.54	84.76±15.89	1.398	0.249
NIHSS on admission^#^	7(4–11.75)	5(3–11.25)	9(6–14)	11.056	0.004

*mean ± standard deviation;

^#^median and interquartiles range

### 3.2 Effect of RFID time management on AIS outcome

Regarding primary outcome, the in-hospital mortality rate was not statistically different among the three groups (deterioration and abandonment of treatment were treated as in-hospital death). Length of hospital stay was significantly reduced in in-hospital and whole process RFID groups. The secondary outcomes, including NIHSS score, mRS score at discharge, and intracerebral hemorrhage, were not significantly different among the groups (see Table 2).

**Table 2:** Comparison of outcomes among the three groups.

Primary outcome	Traditional Group (N = 30)	In-hospital RFID Group (N = 144)	Whole process RFID Group (N = 29)	*z/χ* ^*2*^	P
In-hospital mortality (hospice), yes, n (%)	4 (13.33%)	20 (13.89%)	3 (10.34%)	0.263	0.877
Secondary outcome					
Intracerebral hemorrhage, yes, n (%)	1 (3.33%)	3 (2.08%)	0(0.00%)	0.881	0.644
Hospital stays (days)^#^	10 (7.5–12)	7 (6–10)	7 (4–9)	11.355	0.003
NIHSS score at discharge^#^	2 (0–6)	2 (0–5)	1 (0–3.5)	0.135	0.935
mRS of discharge^#^	2 (0–3)	1 (0–3)	1(1–3)	0.706	0.703

^#^median and interquartiles range

### 3.3 Comparison of time metrics in emergency care process

As shown in Table 3, ODT in the traditional group, in-hospital RFID group, and whole process RFID group were 110.33±63.62minutes, 149.47±85.48minutes, and 101.38±47.05 minutes, respectively. The shortest ODT was observed in the whole process RFID group and longest was in the in-hospital RFID group (F = 6.575, P = 0.002). There was no significant difference between the ODT comparing the traditional group to the whole process RFID group (p = 0.661). Both in-hospital and whole process RFID groups showed a significantly reduced DNT of 33.95±26.80 minutes and 32.59±25.45 minutes compared to the DNT of 125±43.16 minutes in the traditional group (F = 121.857, P<0.001). The OTT was 144.31±47.96 minutes in the whole process RFID group, and 183.35±85.47 minutes in the in-hospital RFID group, both significantly shorter than 235.53±57.27 minutes in the traditional group (F = 10.377, P<0.001).

**Table 3:** Time metrics of patients treated with IVT among the three groups.

	Traditional Group (N = 30)	In-hospital RFID Group (N = 144)	Whole process RFID Group (N = 29)	*χ* ^*2*^ */ F*	P
Onset-to-Call time (min)^#^	71(10.25–113.75)	66(28–117)	60 (39–104)	0.644	0.725
Call-to-Ambulance on scene time(min)^#^	19(15–25)	15(12–19)	11 (10–19)	8.712	0.013
Ambulance on scene-to Door time (min)^#^	17.5(13–22)	32(17–52)	16 (11–22)	31.387	< 0.001
Door-to-CT time (min)^#^	42 (27.5–54.5)	16(13–20)	13 (8–18)	63.783	< 0.001
CT-to-Thrombolysis time (min)^#^	71(51–108)	10(5–18.25)	9 (5–16)	53.887	< 0.001
Onset-to-Door time (min)*	110.33±63.62	149.47±85.48	101.38±47.05	6.575	0.002
Door-to-needle for Thrombolysis time (min)*	125.00±43.16	33.95±26.80	32.59±25.45	121.857	< 0.001
Onset-to-Thrombolysis time (min)*	235.53±57.27	183.35±85.47	144.31±47.96	10.377	< 0.001

*mean ± standard deviation;

^#^median and interquartiles range

### 3.4 Composition of the AIS emergency care time metrics

**Fig 2:**
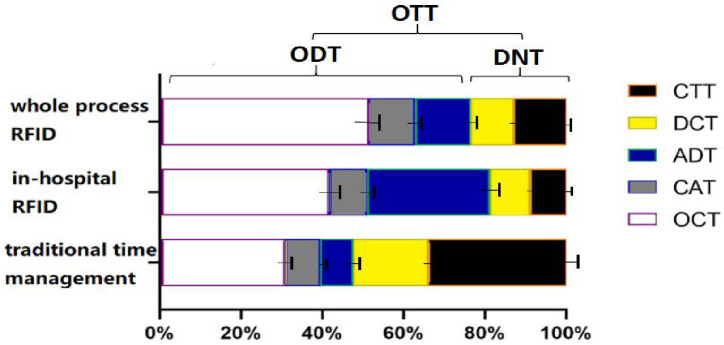
Composition of emergency time metrics. OTT: symptom onset to thrombolysis time; ODT: symptom onset to door time; DNT: door to needle for thrombolysis time; OCT: symptom onset to emergency call time; CAT: emergency call to ambulance time; ADT: ambulance on scene to door time; DCT: door to CT room time; CTT: CT to thrombolysis time.

The composition of the AIS emergency care time metrics is shown in Fig 2. OTT is composed of ODT and DNT, whereas ODT is composed of OCT, CAT, and ADT. DNT is comprised of DCT and CTT. ODT and DNT account for 75.68% and 22.65% of OTT respectively in the whole process RFID group, and 80.83% and 18.52% respectively in the in-hospital RFID group. ODT accounted for 46.84% of OTT in the traditional group, which was significantly lower whereas the percentage of DNT at 53.6% was significantly higher, χ^2^ = 31.643, p<0.001.

## 4. Discussion

The results of the study suggest that after applying in-hospital RFID time management, the in-hospital emergency care time for AIS patients significantly decreased, that is DNT decreased from 125.00±43.16 min to 33.95±26.80 min. After applying the whole process RFID time management, the whole emergency care time was further reduced, that is OTT was decreased from 235.53±57.27 min in the traditional group to 144.31±47.96 min in whole process RFID time management. Compared to the traditional time management system, the adoption of RFID is not only beneficial for reducing DNT of in-hospital procedure, but also for reducing the whole emergency procedure.

Recommendation for early management sets specific time requirements for each step of the AIS emergency care procedure, such as the time from hospital arrival to blood collection, the time from hospital arrival to CT room, ODT, DNT, etc. [6]. Essentially, time management is critical. Stroke centers organize emergency units such as emergency department, CT unit, and neurology to form multidisciplinary teams, to allow more AIS patients to receive IVT treatment in a timely manner [7]. However, multidisciplinary cooperation is not a simple combination of disciplines but involves the continuous integration and optimization of the multidisciplinary workflow [8]. Discovering disconnects in the multidisciplinary collaborative process facilitates continuous quality improvement and reduction in emergency care time [9]. Documentation of emergency time points is thus of critical importance for identifying problems. Inaccurate time recording cannot accurately identify delays in time, thus preventing continuous quality analysis and improvement. Time management is currently commonly applied in stroke centers, and time record sheet is typically adopted to record the time of each step of the standard AIS emergency [10]. In addition, considering the possibility of errors in recording time manually, most stroke centers record time automatically by computer or other automation devices. For example, time is automatically recorded through an electronic information system when medical staff presses an on-screen button [11]. Although this method is convenient for recording time and reduces potential for error, there is a lag in time points recording, leading to unreliable results. That is, the AIS emergency process must prioritize all emergency care operations before logging into the electronic information system to sign and execute medical orders or to issue reports. Moreover, it cannot be excluded that prioritizing time registration in pursuit of time attainment results in actual treatment delays. Therefore, developing an integrated, automated time recording system for time management is an important challenge facing the quality improvement of AIS emergency care [12].

RFID technology, an advanced technique for locating and tracking items, has been used for nearly 50 years [13]. Currently, RFID has been used in the medical field to track the transfer chain of drugs, blood products, consumables, etc. [14]. However, few studies report on the application of the RFID technology in the emergency care of patients with acute and critical conditions, such as stroke patients, to monitor and record the emergency time points to streamline the emergency care process. This study confirmed that the application of RFID technology could accurately and automatically record each time point of the AIS treatment, enabling reliable detections of time-delayed aspects of treatment, thereby targeting specific aspects of the emergency care process and developing quality control measures.

DNT is a major indicator of the efficiency of in-hospital time management, which mainly consists of DCT, and CTT. Currently, in-hospital time management has received sufficient attention, with many studies report on optimization for in-hospital emergency care procedure. The Helsinki model was one of the successful optimizing strategies, which could be used to attain the target of DNT within 25min [15]. A quality improvement system with time management, contributing to streamlining the workflow of the AIS patient, was also proven to reduce DNT to 26min [16]. In the current study, before the application of in-hospital RFID time management, DCT (including blood collection and ECG time) and CTT were longer, indicating that the delay in in-hospital emergency process mainly occurred in the emergency department or CT room, probably related to delayed arrival notification or CT room occupancy, etc. After applying RFID in-hospital time management, DNT was reduced significantly, which suggested the times achieved in the time management. However, the success does not lies in RFID itself, but a continuous appraisal of workflow at all enrolling sites. RFID time management allowed for early identification of potential workflow defects as well as recognition of best solutions. In particular, it allowed for pre-hospital notifications to receive the patient and clearance of the scanner.

ODT primarily reflects the efficiency of the pre-hospital transfer workflow. ODT encompasses the main part of the overall treatment workflow for AIS patients, from first medical contact to thrombolysis. It consists of time from OCT, CAT, and ADT. Few studies focused on optimization for pre-hospital emergency procedure to reduce ODT. Data from 323,601 stroke cases in 1337 hospitals of the Chinese Stroke Center Consortium showed that only 29.3% of AIS patients received IVT within 4.5hours, where the majority of the time was spent on pre-hospital process [17]. Laurent E. [18] showed that the ODT indicators of over 60% AIS cases in France were over 4 hours. Pre-hospital factors resulted in the greatest delay to thrombolysis administration. Long ODT was more common in patients with atypical, or less severe, symptoms, including the elderly, and those living alone [19]. Pre-hospital workflow optimization and time management is of critical importance. In the current study, with the implementation of RFID in-hospital time management, while DNT was reduced, ODT was even longer comparing to traditional time management. This may be related to the fact that when patients arrive, the hospital prepares the in-hospital process before triage to ensure that the DNT meets the standard, thereby causing patients to wait for admission. This problem was avoided with the implementation of the whole process RFID time management system. The RFID technology was extended to pre-hospital phase as early as the arrival of the ambulance, integrating the EMS data with the hospital’s automated information recording system, therefore, the entire emergency care process of AIS patients is recorded in full. However, the ODT was not reduced significantly comparing the traditional group to the whole process RFID group. Other than during a COVID surge that may have increased overall time required for initial EMS evaluation and transportation [20, 21], patient awareness of calling for EMS is an important factor which may result in time delay in pre-hospital. The current study shows that ODT accounts for 75.68% in the entire emergency process, therefore, Further research is needed to optimize pre-hospital emergency care workflow.

### 4.1 Limitations and conclusions

First, the current study is a retrospective cohort analysis. It is not a randomized clinical trial, thus there might be important differences in baseline characteristics, such as NIHSS score, between patients across the three groups based on the type of RFID time management used, which may bias comparisons and outcomes.

Second, the study is a single-center study; the data in the study came from patients treated in the same hospital. It is possible that patients who were at the hospital tended to have more severe strokes, therefore, the generalizability of the findings cannot be over-estimated.

Third, precise information on the time of symptom onset is not available. The symptom onset time is provided by patients from memory. Different patients may provide different onset times because of their different understanding of symptoms.

Fourth, as a retrospective study, no long-term prognostic indicators were obtained, such as 90-day mortality after discharge, 90-day mRS score, etc. Therefore, this study cannot adequately provide information on whether RFID time management can improve patient prognosis.

Fifth, the proportion of AIS patients admitted to the hospital by calling EMS is not high, so the total number of cases in the study is somewhat insufficient, and the sample size should be expanded in future research.

Sixth, Multidisciplinary Collaboration and Workflow Optimization in the study may also play a role for the DNT shortening not just the Radio frequency identification technology.

Overall, we conclude that RFID time management can effectively detect time delays, provide a basis for analyzing causes of delays in order to formulate improvement measures, thereby shortening OTT. The study found that the current RFID time management has achieved remarkable results in promoting the improvement of the hospital’s emergency care workflow. Further, while DNT has been greatly shortened, ODT has not been significantly shortened. Therefore, it remains to be further studied how to optimize the pre-hospital phase of the emergency care process with time management.

### 4.2 Conclusions

Time management with RFID was proved to be effective in reducing DNT, and might also reduce ODT of pre-hospital emergency procedure in AIS patients with thrombolysis treatment, thus OTT of the whole emergency process was reduced.

## Supporting information

S1 Raw data (ZIP)
