# Supplementary material for: Radio frequency identification technology reduce intravenous thrombolysis time in acute ischemic stroke
Source: PLoS One. 2023 Jul 19;18(7):e0288207. doi: 10.1371/journal.pone.0288207 (PMC10355394; doi:10.1371/journal.pone.0288207)
Supplement: Attachment — Submitted filename: response to reviewers.docx [file pone.0288207.s002.docx]

Reviewer #1: The authors performed a retrospective observational cohort study investigating the impact on utilizing RFID technology in acute ischemic stroke patients to reduce the onset to IV thrombolysis time (OTT). There are significant issues that need to be addressed prior to the paper being acceptable for publication.

Abstract:
1. The acronym “OTT” is not adequately defined. The phrase “onset to intravenous thrombolysis time” should be stated prior to using the acronym.
Answer:

It was corrected with red fonts.

Thank you.

Introduction:
1. The authors stated every one minute delay in care can “reduce the patient’s brain cells by 1.9 million and the patient’s life expectancy by one week.” Please provide a reference for this statistic.

Answer:

We confirmed “reduce the patient’s brain cells by 1.9 million”, the reference was added in the section of references with red fonts. Thank you for suggestion.

However, “the patient’s life expectancy by one week” was not confirmed, we decide to delete it, sorry for that.

[]Saver, JL. Time is brain--quantified. STROKE. 2006,37(1):263-266.

2. The sentence “…intravenous thrombolytic therapy should be obtained within 4.5 hours after the onset of AIS” should be revised; the word “obtained” should be changed to “administered.”

Answer:

Thank you. It was corrected with red fonts.

3. The authors refer to the first 70 minutes to OTT as the “golden window.” It is not clear what this phrase means, please clarify or re-phrase.

Answer:

The term golden window means 60 minutes, which has been attributed to the trauma surgeon R Adams Cowley, who recognized that the sooner trauma patients receive definitive care—particularly within the first hour after trauma—the better their chance of survival. To illustrate the pronounced time sensitivity of acute stroke management, the term has also been applied to this clinical situation. Sorry for that, now it was corrected.

Thank you for reminding.

Methods:
1. The sentence “Subsequently, the RFID technique is extended…” should be modified so the word “is” is replaced with “was.”

Answer: thank you for reminding. It was corrected with red fonts.

2. The initial management steps listed between the three groups is inconsistent. For instance, the traditional group mentions that an electrocardiogram and blood samples are obtained, but this information is not listed in the two RFID groups. Were these steps eliminated? Also, only the second group states that a stroke physician makes the decision regarding administration of thrombolysis. Please clarify if the stroke physician (or another physician) makes the decision to administer thrombolysis in each group.

Answer:

Thank you for suggestion. It were corrected with red fonts.

Electrocardiogram and blood samples is performed when patient arrive.

The decision to administer thrombolysis is decided by stroke physician after CT scan.

3. Patient Selection, Inclusion: Which “diagnostic criteria according to the 2018 guidelines” are you referring to?

Answer:

All patients conform to the diagnostic criteria according to the AHA/ASA guidelines (guidelines)[4] as follows: (a) acute onset; (b)with symptoms or signs of neurological deficits; (c)confirmation of AIS on brain CT/MRI; (d) exclude cerebral hemorrhage and non-vascular causes.

It was corrected with red fonts. Thank you for suggestion.

4. Were patients who presented within the 4.5 hour time window for thrombolysis but did not receive it due a delay in evaluation included in the study (if there were any)? For instance, if a patient presented within 3 hours of symptom onset, but evaluation was not completed until after 4.5 hours, was this patient accounted for in the study?

Answer:

According to recommendation of AHA/ASA, patients received intravenous thrombolysis after 6 hours from symptom onset were excluded.

Thank you for reminding. It was corrected with red fonts in Method section of Patient selection.

5. Statistical Analysis: The sentence “Categorical data are expressed as numbers…” should be modified so that the word “numbers” is changed to “frequency.”
Answer:

Thank you for suggestion. It was corrected now.

Results:
1. The tables are difficult to read as presented. Currently, all dichotomous variables are presented as yes/no with a frequency for each. It would be much easier to read the table if only the number of “yes” variables are included followed by the percentage of the overall cohort (for example, hypertension n=21 (70%). This should be applied to all dichotomous variables.

Answer:

It was corrected as your suggestion, thank you.

2. Table 1: Age and blood pressure readings are followed by +/-XX. Please clarify if these numbers are standard deviations.

Answer:

It is mean ± standard deviation, and it was marked as foot note following the table. Thank you.

3. Table 1: NIHSS is followed by numbers in parenthesis. Does this represent range? Interquartile range? Please clarify.

Answer:

It represents interquartile range, and it was marked as foot note following the table.

Thank you.

4. Section 3.2: The authors report in-hospital mortality as the primary outcome. This was not mentioned as an outcome in the methods. The last sentence of the introduction states the study compares OTT and the effect of RFID on time management. Please clarify.

Answer:

It was added as outcome in Methods.

The study compares OTT before and after RFID to inspect the effect of RFID on time management and patient outcomes. It was corrected in Introduction.

Thank you.

5. Table 3: What do these numbers represent? Mean? Median? Range? Interquartile range? The table and the text need to be revised to clarify.

Answer:

It represents median and interquartile range, and it was marked as foot note following the table.

Thank you.

6. Section 3.3: The first sentence needs to be re-written. The authors use the word “respectively” to describe the order of the groups and ODT times, but this is incorrect.

Answer:

The first sentence was re-written with red fonts.

Thank you.

7. Section 3.3: The p-value listed for ODT times in the text does not match the value listed in the table. Which is correct?

Answer:

The table is correct. Sorry for that.

It was corrected.

Thank you.

8. Section 3.4: The authors state OTT is composed of OTD and DTN times. The summation of OTD and DTN is not equal to OTT listed in table 3. Please comment on these discrepancies or correct the data.
Answer:

OTT is composed of ODT and DNT. Now it was corrected and the summation of ODT and DNT is equal to OTT now.

Thank you for reminding.

Discussion:
1. “DNT decreased from 118min to 26min” and “OTT was reduced to 110min from 200min…180min” This does not match Table 3, please correct.

Answer:

It was corrected.

Thank you.

2. The authors state the adoption of RFID not only decreased DNT, but also reduced ODT. While the mean ODT is lowest in the whole process RFID group, it is only 9 minutes shorter than the mean ODT in the traditional group. I question if a confounding factor in the in-hospital RFID group is skewing the data (was this during a COVID surge that may have increased overall time required for initial EMS evaluation and transportation, or some other factor)? Was there a significant different between the ODT comparing the traditional group to the whole process RFID group?

Answer:

Indeed, there was no significant difference between the ODT comparing the traditional group to the whole process RFID group. In addition to confounding factors (a COVID surge), patient awareness of calling for EMS is an important factor influencing emergency time in pre-hospital. These was proved by the study, which found that ODT accounts for 75.68% in the entire emergency process, and OCT(symptom onset to call for EMS time) is a main component of it.

All these were corrected in the section of Discussion with red fonts.

Thank you.

3. The authors suggest delayed arrival notification or CT room occupancy were factors contributing to delays in OTT. Can the authors comment on specifically how RFID was used the make improvements in this time management process? If a quality improvement initiative was performed implementing data collected with RFID technology, it would be useful to discuss the specifics of such a process.
Answer:

Yes, a quality improvement initiative was performed implementing data collected with RFID technology. We discuss it as follows with red fonts in section of Discussion:

After applying RFID in-hospital time management, DNT was reduced significantly, which suggested the times achieved in the time management. However, the success does not lies in RFID itself, but a continuous appraisal of workflow at all enrolling sites. RFID time management allowed for early identification of potential workflow hurdles as well as recognition of best practices. In particular, it allowed for pre-hospital notifications to receive the patient and clearance of the scanner.

Conclusion:
1. The authors state RFID was effective at reducing both DNT and ODT. I question whether the data supports the statement that RFID reduced ODT. Furthermore, the authors state in the limitations that RFID reduced DNT, but ODT was not significantly shortened, contradicting the statement in the conclusion.

Answer:

Sorry for the contradicting statement. Since some confounding factors, the ODT decreased, however not significantly, in the whole process RFID group. It was corrected in the conclusion with red fonts.

Thank you for reminding.

Reviewer #2: Thank you very much for inviting me to review this manuscript. In this manuscript, the authors retrospectively analyzed patients with AIS, transferred by Emergency Medical Services (EMS) to Hunan Provincial People's Hospital between September 2019 to June 2022. Authors concluded that: Time management with RFID is effective in reducing OTT in AIS patients with thrombolysis treatment. I have some suggestions to improve the manuscript.
1.Introduction: ‘Every one-minute delay in the patient’s AIS emergency care procedure can reduce the patient’s brain cells by 1.9 million, and the patient’s life expectancy by one week’ authors should provide the citation.

Answer:

The citation was added in the section of references with red fonts. Thank you for suggestion.

2.Introduction : The previous study has demonstrated that the thrombolysis time and treatment time can be shortened by Multidisciplinary Collaboration and Workflow Optimization PMID: 34735901; PMID: 35883030

Answer:

Yes, it was proved that multidisciplinary collaboration and workflow optimization result in shorten intravenous thrombolysis time, and time management would facilitate the multidisciplinary collaboration and workflow optimization by identifying delays of workflow in AIS care.

It was added in red fonts, thank you.

3.2.2 Patients Selection The authors should provide the citation of patients selection or more explanations

Answer:

All patients conform to the diagnostic criteria according to the AHA/ASA guidelines (guidelines) as follows: (a) acute onset; (b)with symptoms or signs of neurological deficits; (c)confirmation of AIS on brain CT/MRI; (d) exclude cerebral hemorrhage and non-vascular causes.

The citation is as follows:

[6]Powers, WJ; Rabinstein, AA; Ackerson, T; Tirschwell, DL; et al. Guidelines for the Early Management of Patients With Acute Ischemic Stroke: 2019 Update to the 2018 Guidelines for the Early Management of Acute Ischemic Stroke: A Guideline for Healthcare Professionals From the American Heart Association/American Stroke Association. STROKE. 2019-12-01;50(12):e344-e418.

It was corrected with red fonts. Thank you.

4.3. RESULTS any data was missing?

Answer:

Data of the section of “Composition of the AIS Emergency Care Time Metrics” was added as you can see in Fig. 2.

Thank you.

5.Table 1 ‘Demographic characteristics’ was very odd in the second line ,please move it to the first line. And same problem exist in table 2

Answer:

It was corrected.

Thank you.

6.Please provide the citations of DISCUSSION part

Answer:

Citations were added.

Thank you.

7.DISCUSSION, because the study was across the covid19 period, PMID: 36257718 PMID: 36124298, did the covid19 affected the study?

Answer:

It may be affected by the COVID19 wave, and it was added in section of Discussion with red fonts. And the citation was added too.

Thank you.

8.Limitations and Conclusions: Multidisciplinary Collaboration and Workflow Optimization may also play a role for the DNT shortening not just the Radio frequency identification technology.

Answer:

It was added in the section of Discussion.

Thank you.

PLOS authors have the option to publish the peer review history of their article ([what does this mean?](https://journals.plos.org/plosone/s/editorial-and-peer-review-process#loc-peer-review-history)). If published, this will include your full peer review and any attached files.


If you choose “no”, your identity will remain anonymous but your review may still be made public.
No

Thank you.

**Do you want your identity to be public for this peer review?** For information about this choice, including consent withdrawal, please see our [Privacy Policy](https://www.plos.org/privacy-policy).

Reviewer #1: No

Reviewer #2: No
